# New insights into microstructure of irradiated beryllium based on experiments and computer simulations

**DOI:** 10.1038/s41598-020-64654-5

**Published:** 2020-05-15

**Authors:** M. Klimenkov, P. Vladimirov, U. Jäntsch, V. Kuksenko, R. Rolli, A. Möslang, N. Zimber

**Affiliations:** 1 Karlsruhe Institute of Technology (KIT), Institute for Applied Materials - Applied Materials Physics (IAM-AWP), Hermann-von-Helmholtz-Platz 1, 76344 Eggenstein-Leopoldshafen, Germany; 2 Culham Centre for Fusion Energy, Culham Science Centre, Abingdon, Oxfordshire OX14 3DB United Kingdom; 3 Karlsruhe Institute of Technology (KIT), Institute for Applied Materials - Materials and Biomechanics (IAM-WBM), Hermann-von-Helmholtz-Platz 1, 76344 Eggenstein-Leopoldshafen, Germany

**Keywords:** Energy science and technology, Nuclear energy, Nuclear fusion and fission

## Abstract

The microstructural response of beryllium after neutron irradiation at various temperatures (643–923 K) was systematically studied using analytical transmission electron microscope that together with outcomes from advanced atomistic modelling provides new insights in the mechanisms of microstructural changes in this material. The most prominent feature of microstructural modification is the formation of gas bubbles, which is revealed at all studied irradiation temperatures. Except for the lowest irradiation temperature, gas bubbles have the shape of thin hexagonal prisms with average height and diameter increasing with temperature. A high number density of small bubbles is observed within grains, while significantly larger bubbles are formed along high-angle grain boundaries (GB). Denuded zones (DZ) nearly free from bubbles are found along both high- and low-angle grain boundaries. Precipitations of secondary phases (mainly intermetallic Al-Fe-Be) were observed inside grains, along dislocation lines and at GBs. EDX analysis has revealed homogeneous segregation of chromium and iron along GBs. The observed features are discussed with respect to the available atomistic modelling results. In particular, we present a plausible reasoning for the abundant formation of gas bubbles on intermetallic precipitates, observation of various thickness of zones denuded in gas bubbles and precipitates, and their relation to the atomic scale diffusion mechanisms of solute-vacancy clusters.

## Introduction

Being lightweight metal, beryllium finds numerous technological applications ranging from aerospace and nuclear industry to mobile phones due to its exceptional physical properties such as high strength, electrical conductivity and high melting point. It has an anisotropic hexagonal close packed crystal lattice structure, which controls the complex properties of irradiation-induced self-point defects and dissolved foreign atoms^1,2^. Their dynamic interplay with dislocations and grain boundaries (GBs) determines the microstructure evolution under irradiation. Therefore, investigations of beryllium microstructure after irradiation allow not only qualification of material properties for its prospective nuclear fusion reactor applications^3^, but also contribute significantly to the physical understanding of the underlying mechanisms of microstructural changes in hexagonal closed packed metals under irradiation or high temperature aging in general. In addition, such investigations provide by analogy better understanding of the microscopic processes occurring in materials with other lattice structures.

In nuclear fusion technology, beryllium is considered as a “First Wall” material in ITER, presently one of the largest mankind projects worldwide^3–5^, but also as a neutron multiplier material in different tritium-breeding blanket concepts for the future demonstration fusion power plant DEMO^6,7^. In one of the blanket concepts, called helium-cooled pebble bed (HCPB), interchanged layers of a lithium ceramic and beryllium pebbles will be used. The pebbles are expected to be exposed to high-dose irradiation by energetic neutrons resulting in considerable irradiation damage and generation of transmutation induced helium (^4^He) and tritium (^3^H) over the years of operation of the fusion reactor. In the present HCPB blanket design, the maximum operating temperature of beryllium pebble beds can reach 923 K (650 °C)^8^.

Long-term irradiation tests in material research reactors provide important information about irradiation resistance of beryllium pebbles under close-to-fusion conditions regarding operation temperature, accumulated damage dose, amount of helium and tritium generated by neutron-induced transmutation, although the energy spectrum of fusion neutrons cannot be exactly simulated by common nuclear reactors. Previous beryllium irradiations were performed either at rather low temperatures (70–400 °C)^9,10^ and high fast neutron fluence (up to 10^23^ cm^−2^) or at higher temperatures (>500 °C) and relatively low fluence (<2·10^22^ cm^−2^)^11–16^. To simulate fusion irradiation conditions closely, both high irradiation temperatures (450–650 °C) and high neutron fluences are required. The irradiation campaign HIDOBE, whose results are reported here, covers the previously missed region of high temperature 370–650 °C (643–923 K) and high neutron fluence (up to 3·10^22^ cm^−2^) being especially relevant for the fusion reactor applications of beryllium. The lack of data in this range emphasizes the importance of such irradiation campaigns like HIDOBE as a unique source of information on beryllium for both blanket design and general scientific understanding of underlying atomic scale processes.

This paper presents systematic analytical transmission electron microscopy (TEM) studies of the microstructural changes in neutron irradiated beryllium pebbles reaching a damage dose and gas production of 37 dpa, 5900 appm ^4^He and 640 appm ^3^H, respectively. These experimental results are discussed based on *ab initio* simulations of solute-vacancy interactions, migration energies for solute atom diffusion and their implications for solute migration mechanisms and anisotropy of denuded zones.

## Results

### Microstructure of as received beryllium

**Figure 1:**
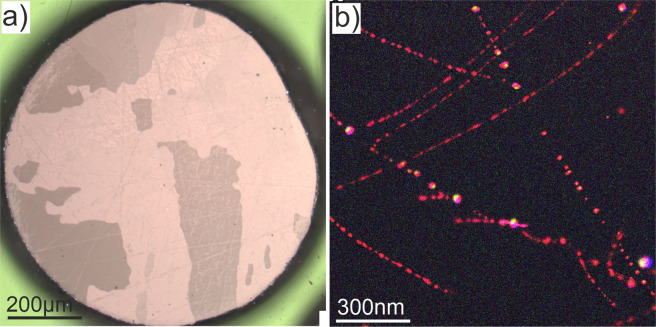
The light microscopy image of a 1 mm pebble (**a**) and TEM-EDX map an intra-grain area (**b**).

The typical microstructure of 1 mm beryllium pebbles produced by rotating electrode process^17^ is shown in Fig. 1. The polished cross-section through the center was recorded with a polarization microscope depicting various grain orientation by color changes (Fig. 1a). As far as pebbles represent molten beryllium droplets solidified on the fly by cooling in inert gas atmosphere, they often show the radial grains grown starting from the pebble surface towards the its center during rapid cooling. The size of the grains varies in a wide range: from 2–3 µm to 100–200 µm. Normally 10 to 60 grains could be detected within typical cross-section.

Line dislocations inside grains are normally decorated with different beryllide phases formed from impurity atoms. The EDX map was taken with Fig. 1b shows for example the distribution of Fe-Al-Si phase with red, Cr-Al-Si-Mn with yellow and Al-Mg with blue colors, respectively. Observed precipitates show often a complex, multi-phase composition. We assume that such peculiar distribution of precipitates depends on the local concentration of impurities which prefer to accumulate inside dislocation cores.

### Bubbles inside grains

**Figure 2:**
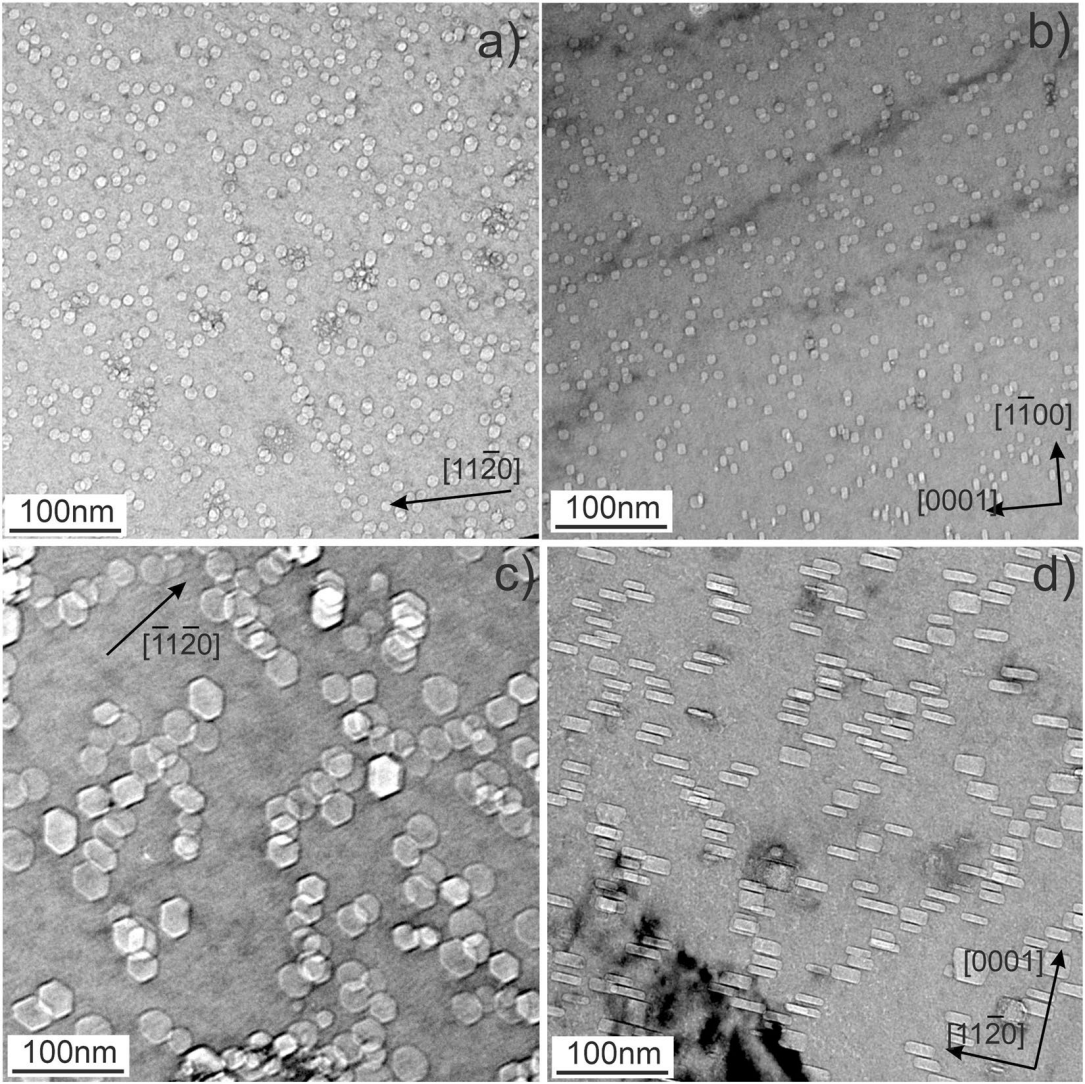
Gas bubbles in beryllium pebbles irradiated at 643 K (**a,b**) and at 713 K (**c,d**). The areas imaged in parts “a” and “c” are oriented near to the *(0001)*_*h*_ zone axis and in parts “b” and “d” they are oriented along the zone axis perpendicular to the *(0001)*_*h*_ direction.

**Figure 3:**
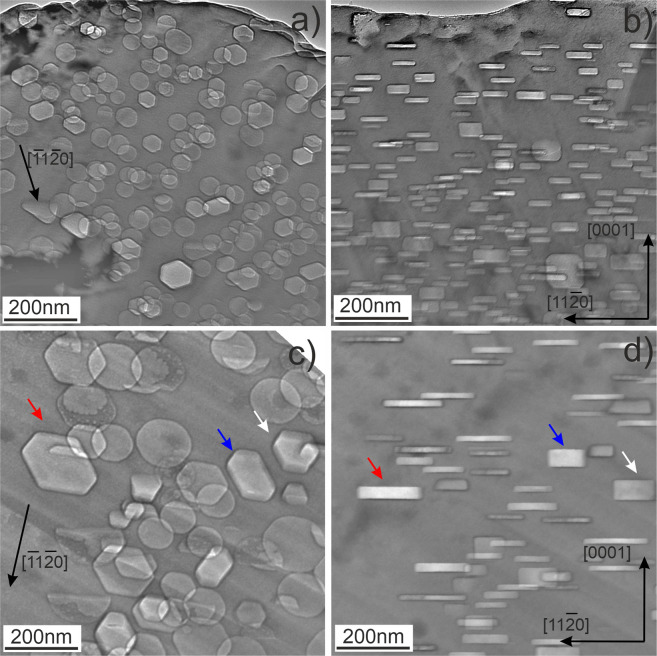
Gas bubbles in beryllium pebbles irradiated at 833 K (**a,b**) and 923 K (**c,d**). The areas imaged in parts “a” and “c” are oriented near to the *(0001)*_*h*_ zone axis and in parts “b” and “d” they are oriented along the zone axis perpendicular to the *(0001)*_*h*_ direction. Note that thicker bubbles have more pronounced faceting (see bubbles marked with arrows in 2c, d).

Typical for beryllium irradiated at elevated temperatures intragrain microstructures containing gas bubbles are illustrated in Figs. 2–3 for all studied irradiation temperatures. The bubbles observed after irradiation at the lowest irradiation temperature of 643 K (370 °C) (Fig. 2a) have a size of ca. 10 nm with a round or slightly faceted shape. Tilting of the lamellae does not reveal any anisotropy in the shape of bubbles inside grains, suggesting that their shape is close to spherical.

In contrast to the lowest temperature, bubbles grown at higher temperatures have a pronounced hexagonal prismatic shape with their bases located on basal crystallographic planes $\{0001\}$ (Fig. 2c,d, Fig. 3).

**Figure 4:**
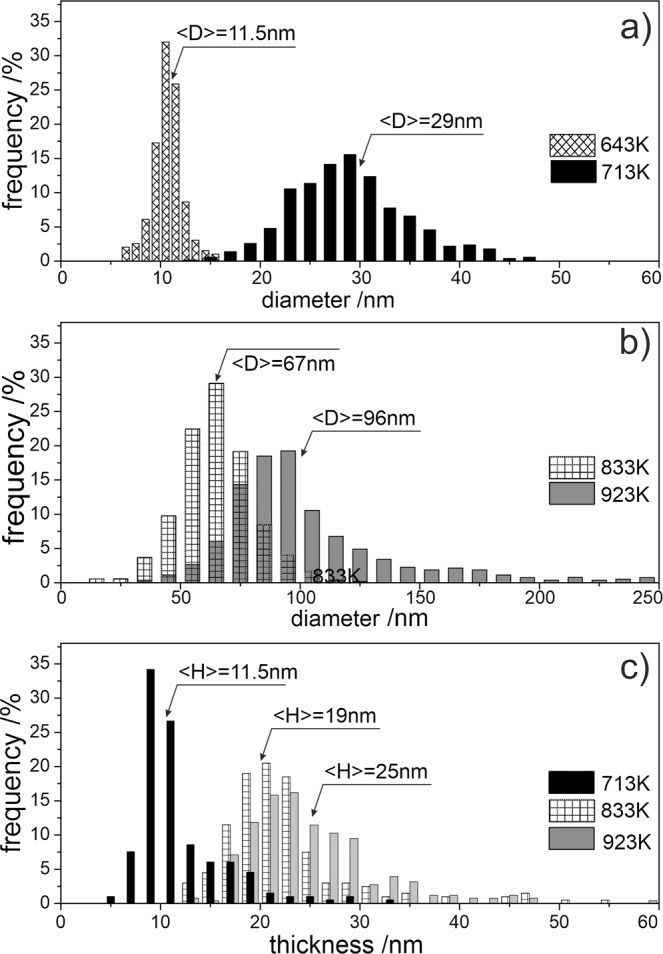
Distribution of diameter (**a,b**) and thickness (**c**) of bubbles measured at various irradiation temperatures.

Figure 4 shows statistical distributions of diameter (a, b) and thickness (c) of the bubbles for all irradiation temperatures. The bubbles formed at 643 K (370 °C) obey a narrow size distribution with a mean diameter of 11.5 nm. Due to the round shape of the bubbles, no distinction between diameter and thickness was made for this irradiation temperature (Fig. 4a). As the bubbles formed above this temperature have a hexagonal coin-like shape, both distribution of diameter and height were measured using different lamellae orientations. Approximately 10–15% of bubbles have thicknesses more than two times larger than the average. The statistical data are summarized in Table S1.

### Microstructure at grain boundaries

**Figure 5:**
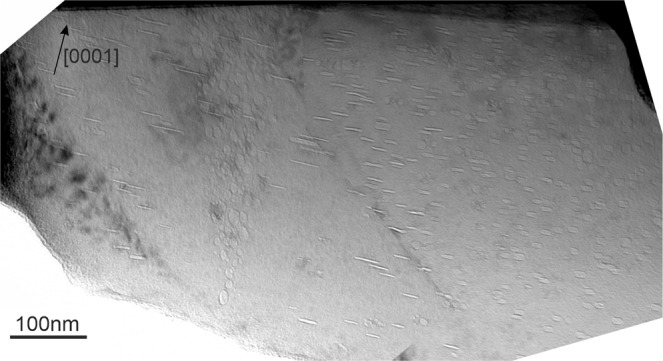
A low-angle (<5°) GB within the pebble irradiated at 643 K as imaged in TEM in bright-field (BF) mode.

**Figure 6:**
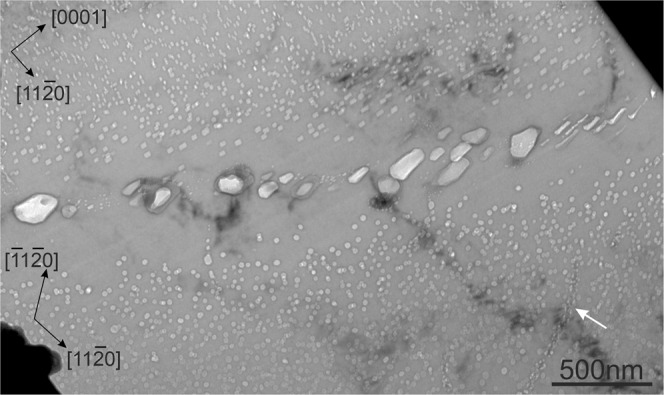
A high-angle GB with misorientation angle of ~90° in a pebble irradiated at 713 K. Dislocation line decorated by Fe-Al-Be precipitates and gas bubbles is marked by arrow.

**Figure 7:**
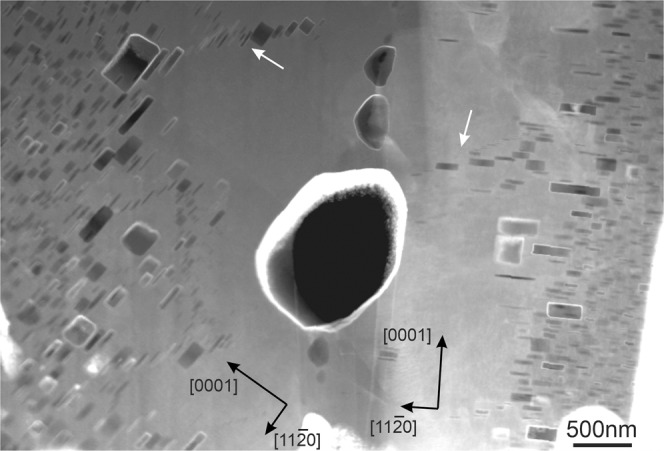
A high-angle GB with misorientation angle of ~60° in a pebble irradiated at 923 K. Chain of bubbles ordered presumably along the former dislocation lines is marked by arrow.

Bubbles at GBs manifest significantly larger sizes than those grown within grains (Figs. 5–7). Near GBs bubble-denuded zones containing only a few bubbles are systematically observed.

**Figure 8:**
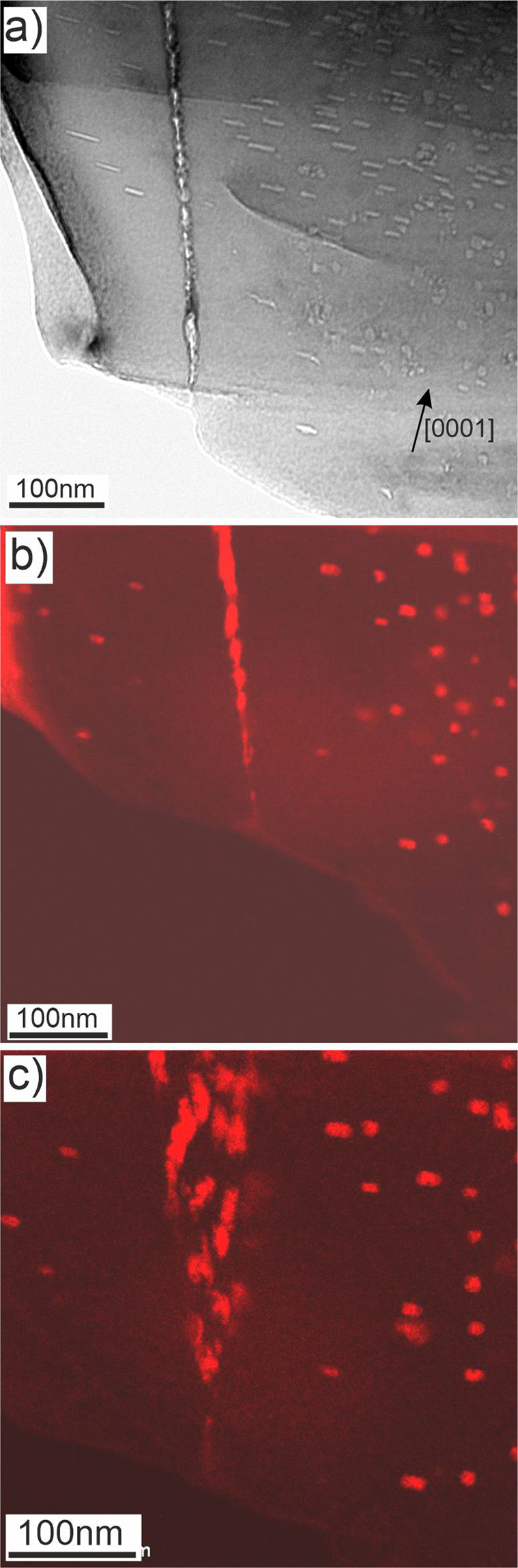
The same low-angle (<5°) GB as shown in Fig. 4 oriented parallel to the electron beam within the pebble irradiated at 643 K (**a**) previously shown in Fig. 4. Two EDX maps (**b,c**) display distributions of Fe-Al-Be particles (colored red) on and around GB. The EDX maps in images (**b**,**c**) are obtained from the same area with ~20° tilt.

An example of low-angle (<5°) GB within the pebble irradiated at 643 K (370 °C) is shown in Fig. 5. The lamella in this area is wedge-shaped so that the GB inclined by ~20° to the electron beam looks like a dense triangular agglomeration of bubbles. After tilting, the same boundary is visible in Fig. 8a as a thin vertical strip surrounded by bubble-denuded zones of ~100 nm.

The width of zones denuded of gas bubbles increases with irradiation temperature (see Table S1) being 350 nm in the pebbles irradiated at 713 K (440 °C) (Fig. 6) and 1200 nm after irradiation at 923 K (650 °C) (Fig. 7). In the latter case, the bubbles have sizes up to 1500 nm.

The bubbles in the region next to the denuded zone show notably larger diameter and thickness than those inside grains (Figs. 6,7). Surprisingly, in the upper grain shown in Fig. 6, the thickness of bubbles adjacent to the denuded zone is increased significantly resulting in a change of the bubbles shape. No pronounced changes of bubble diameter can be observed in the lower grain having another lattice orientation. In several cases, chains of bubbles ordered presumably along former dislocation lines were observed (marked by white arrows in Figs. 6 and 7).

### Second-phase precipitates

TEM study of as-received beryllium pebbles did not reveal any precipitates. After irradiation, spherical second-phase precipitates formed at GBs and inside grains are observed at the two lowest (643 K and 713 K) irradiation temperatures only.

EDX 2D-mappings reveal that spherical precipitates of Fe-Al-Be phase with a size of 10–15 nm are observed within grains of pebbles irradiated at the lowest temperature of 643 K (370 °C), but non-existent within a precipitate-denuded zone with a width of ∼150 nm around the GB (Figs. 5 and 8). As far as distribution of Al follows that of iron, only one color was used for imaging of Al-Fe phase in Fig. 8b,c.

**Figure 9:**
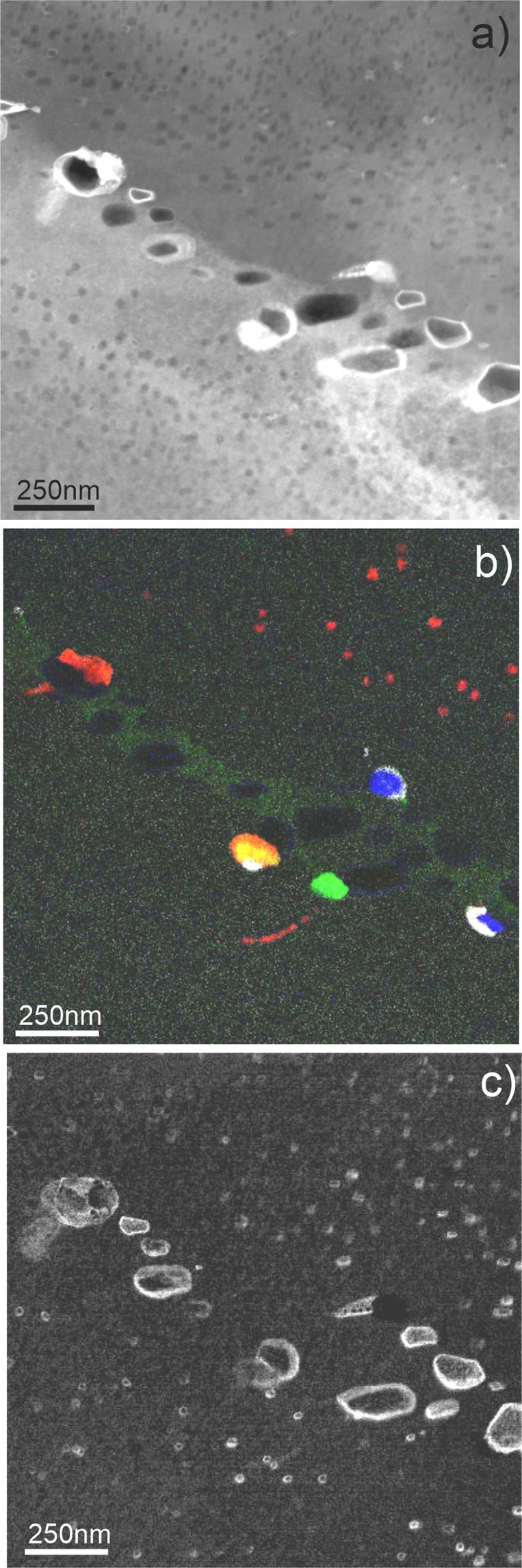
GB with misorientation angle of ~90° in a pebble irradiated at 713 K. Phases in EDX map (**b**) are colored according to the following code: Al-Fe-Be (red), Mg-Si (blue), Cr-Ti (green), Al-Fe-Mn (yellow) and U-Fe (white). Spatial distribution of oxidized open bubbles is shown in image (**c**).

In the pebbles irradiated at the second lowest temperature of 713 K (440 °C), several single- (Ti-Cr) and multi-phase (Fe-Al-Cr-Ti-U and Si-Mg-U) precipitates are found in addition to the Fe-Al-Be phase (Fig. 9). Obviously, uranium forms an outer shell around Si-Mg phase. Homogeneous segregation of chromium and iron (green) along an inclined GB is observed (Fig. 9b). In this figure, all large precipitates are located at the GB, while small 10–15 nm Fe-Al-Be precipitates are in the grain interior. DZs free of precipitates on both sides of the GB can be clearly discerned.

**Figure 10:**
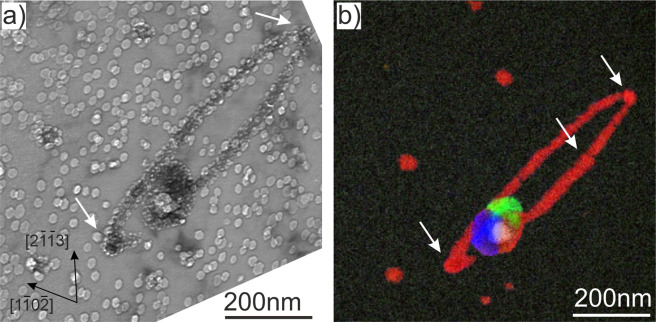
Dislocation loop decorated by a segregation of Fe-Al-Be phase pinned by a complex phase precipitate (**a**) observed at 643 K (370 °C). Various phases in EDX map (**b**) are colored as follows: Al-Fe-Be (red), Mg-Si (blue), Cr-Ti (green) and U-Fe (white).

Sometimes, Fe-Al-Be precipitates were aligned in chains presumably along dislocation lines (Fig. 9) or loops (Fig. 10). In all cases, the surface of Fe-Al-Be precipitates is abundantly decorated with small bubbles of 3–7 nm size. The ring feature and small particles observed in beryllium pebbles irradiated at 713 K consist of Fe-Al-Be intermetallic phase (colored red in Fig. 10b). The 150 nm particle in the middle consists of four phases Fe-Al-Be (red), Mg-Si (blue), Cr-Ti (green), Fe-Al-Mn (yellow) and U-Fe (white). The latter is visible in Fig. 10b as a bright spot in the middle of the particle.

### Swelling

Both, the average bubble diameter *<D* > and the height *<H* > increase with irradiation temperature, while diameter to height ratio *<D* > */* < *H* > rises from one at 643 K (370 °C) for spherical bubbles to approximately four at 923 K (650 °C) (see Table S1) indicating that above 643 K, bubbles grow faster along basal plane than along the c-axis. The dependence of the <D > / < H > ratio on irradiation temperature cannot be explained using construction of thermodynamic equilibrium shape, as in this case the ratio is proportional to the ratio of surface energies on basal and prismatic planes. A kinetic nature of bubble growth or helium pressure should be taken into account for proper explanation.

The mean distance between bubbles *L* was estimated using their number density and assuming for simplicity a spherical shape of the influence zone around each bubble (see Table S1). For the two lowest irradiation temperatures of 643 and 713 K (440 °C), *L* is approximately two times larger than the average diameter *<D* > of the bubbles. Starting from 833 K (560 °C), *L* is already comparable to *<D* > and at 923 K (650 °C) is even smaller than *<D* > . This suggests notable probability of overlapping between neighboring bubbles growing at high irradiation temperatures and their coalescence, tentatively explaining the observed long tails (exceeding twice the average values) in diameter and height distributions of bubbles (see Fig. 4b,c).

The calculation of the volumetric material swelling is essential for the estimation of the radiation influence on mechanical stability of pebbles. The microscopic swelling STEM was calculated from the bubble dimensions measured in the TEM according to the following formula:

$${S}_{TEM}=\frac{\pi H{\sum }^{}{D}_{i}^{2}}{4V}$$

where *D*_*i*_ is the diameter of a bubble, <*H* > the average height of the bubbles, and *V* is the volume where the bubble sizes and their number were measured. The thickness of the area used for the swelling calculations was measured by EELS. Due to the hexagonal prismatic shape of bubbles, both their diameters and thicknesses should be determined simultaneously for more accurate swelling estimation. As far as such detailed information was not collected we multiplied the sum of squares of bubble diameters with the average height <H >.

As shown by Fedorov *et al*.^18^ the formation of bubbles also lead to the microscopic swelling of the material, so that macroscopic swelling should be calculated relative to the initial volume V_0_, instead of the volume of irradiated material V The following correction factor was applied:

$$S=\frac{{S}_{TEM}}{1-{S}_{TEM}}$$

With few percent swelling, this correction is within the range of the measurement error. This correction becomes essential for the swelling values >5%.

**Figure 11:**
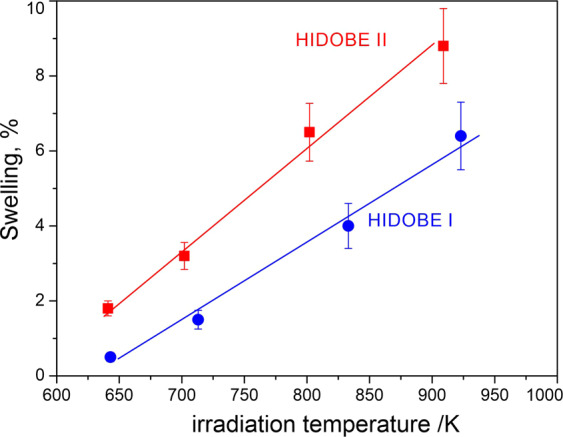
Swelling of 1 mm beryllium pebbles irradiated in HIDOBE-01 (T_irr_ = 641–909 K, D = 13–19 dpa)^2^ and HIDOBE-02 (T_irr_ = 643–923 K, D = 21–37 dpa).

As can be seen from Fig. 11, swelling increases linearly with irradiation temperature for both irradiation campaigns. In spite of the fact that the second campaign was twice as long as the first^19^, the swelling values for HIDOBE-02 are not always twice as big as those obtained from HIDOBE-01 (Fig. 11). At the lowest irradiation temperature, the ratio of swelling values is higher than 2.3 while it reduces to about 1.4 above 700 K.

It should be mentioned, that in reactor different irradiation temperatures were obtained at different vertical positions of drums containing samples with respect to the reactor core. Therefore, not only temperatures differ for these drums, but also neutron flux, and hence, helium and tritium production (see Table 1). Thus, it is instructive to plot the swelling rate measured in percent per accumulated displacement damage, or relative to the helium production. Swelling rate depends also linearly on irradiation temperature and varies from 0.04 to 0.4%/dpa being more than two times lower then often refereed value of 1%/dpa for stainless steel and being smaller or comparable with the value of 0.2%/dpa characteristic for ferritic and ferritic-martensitic steels^20^. Both swelling per dpa and per appm He have the same inclination for both, HIDOBE-01 and −02. Due to elevated irradiation temperatures, swelling per appm He in HIDOBE (~2.7% per 1% He) is notably higher even at the lowest temperature than typical value for low temperature swelling due to helium in solid solution (cf 1.19% per 1% He^9^).

**Table 1:** Irradiation parameters of Ø 1 mm beryllium pebbles from HIDOBE-02^7^.

Irradiation temperature, K			Neutron fluence, 10^25^ m^−2^		D, dpa	^4^He, appm	^3^H, appm
Target	Average	Maximum	Thermal	Fast, ˃1 MeV			
648	643	683	7.89	10.6	21	3632	367
743	713	750	9.87	14.3	29	4751	502
863	833	873	11.3	16.8	34	5524	596
948	923	955	12.0	18.1	37	5925	644

The macroscopic swelling obtained from the dimension measurements show a good correlation to our results at the 643 K and 713 K irradiation temperatures^18^. At 833 K and 923 K temperature the swelling value is 16% and 21%, which is significantly higher than observed in the TEM. The reason for this deviation is the increased formation of micrometer-sized bubbles at grain boundaries, which are hardly visible in the TEM. It has also been shown that beryllium irradiation with Ar ions leads to the formation of a spherical bubble and a high local swelling of about 4%^21^.

### First principle simulations of impurities in beryllium

Previous first principles studies of vacancy clusters^22^ revealed a kind of anomaly in beryllium. In fact, two or more vacancies put together do not form a vacancy cluster, but rather repel each other. Such behavior seems to be in contradiction with a bunch of the experimental data, including the results from the previous section, showing that beryllium swells after neutron irradiation at elevated temperatures^19^. As was shown by us previously^2^, this discrepancy can be resolved by accounting for gas atoms (helium and tritium) generated by neutron-induced nuclear transmutations. These gaseous impurities effectively stabilize vacancy clusters suggesting that bubble nucleation in beryllium occurs heterogeneously, i.e., with a help of nucleation sites e.g., immobile helium atoms at substitutional position. Other, in particular oversized, impurities might be expected to work as nucleation sites for helium bubbles as well. Therefore, the observation of helium bubbles abundantly decorating Fe-Al-Be precipitates reported above inspired us to study properties of major impurities in beryllium by *ab initio* methods.

To study the effect of impurities on heterogeneous nucleation of voids in beryllium, we performed calculations of vacancy binding energies with substitutional solute atoms (Al, Si and Fe) at various distances using *ab initio* approach.

**Figure 12:**
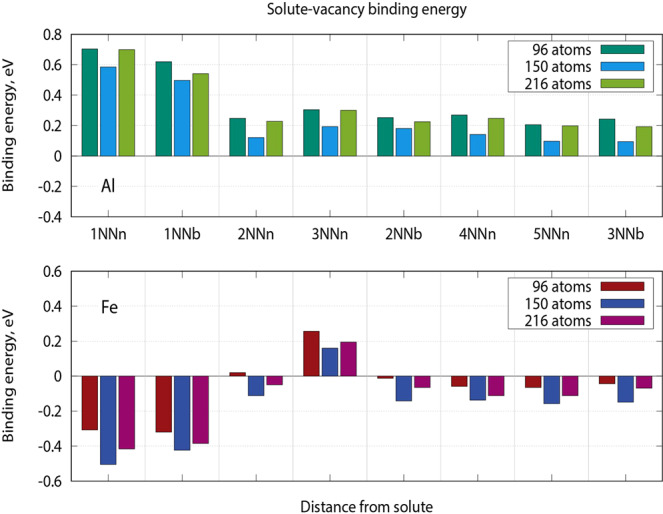
Solute-vacancy binding energies for aluminum (upper plot) and iron (lower plot) for various distances between solute and vacancies. Distances are sorted in the increasing order corresponding the common numbering of nearest neighbors (NN) in hcp lattice and labelled as xNNy where x = 1–5 is an index of nearest neighbor counted either in basal plane (y = b) or outside of basal plane (y = n).

In contrast to our expectations, the first principles calculations revealed repulsion between substitutional iron atom and a vacancy at any distances (see Fig. 12 and Supplementary Table S2), except slight attraction along c-axis (denoted “3NNn” in Table S2) related to the insufficient simulation cell sizes along *c* direction. The repulsion from vacancy is rather unexpected for such, in principle, oversized substitutional atom as iron (atomic radii of Fe is 156 pm cf. 112 pm for Be, see Table S2). However, the changes of the nearest neighbor (NN) distances around Fe-solute calculated with respect to the ideal crystal lattice are quite small: 0.015% expansion of the bond directed to the first NN out of basal plane (1NNn) and −0.023% contraction of the bond to the NN within basal plane (1NNb). The deformation pattern around Al-solute is, however, quite different. In this case, expansion of about 4% was found in both directions. Consequently, aluminum readily attracts vacancies, which reduce considerable lattice expansion around it. The highest binding energies are between aluminum and vacancy sitting on the first NN site out of (1NNn–0.7 eV) and within (1NNb – 0.6 eV) basal plane, respectively (see Table S2). The binding energies for more distant neighbors are below 0.2 eV decreasing rapidly with the distance from the Al-solute. Calculations for silicon solute reveal its even stronger binding with vacancy: about 1 eV and 0.8 eV for the first neighbors out of and in basal plane, respectively. Deformation around Si is smaller than for Al and comprises about 3% in both directions. Surprisingly, substitutional helium with its relatively small atomic radius of 31 pm generate notable expansion in its first neighbor shell: 1.7% and 3.9% for the 1NNn and 1NNb directions, respectively.

Based on these results, it can be concluded that besides helium, also aluminum and silicon can play an important role in gas bubble nucleation in beryllium, while iron tends to repel vacancies and hence cannot serve as a nucleation center for vacancy cluster.

Another important question, which can be elucidated by *ab initio* simulations, is identification of a diffusion mechanism of solute atoms in beryllium. It is reasonable to assume that oversized impurities migrate by vacancy mediated diffusion assuming their strong mutual binding. By analogy with helium behavior in beryllium^1^, one may suppose that oversized solutes also occupy the center of a divacancy thus forming so-called solute-centered divacancy^23^. In this case, solute migrates as a solute-divacancy cluster and, after precipitation of a solute atom, the second vacancy would become available for gas bubble nucleation and growth.

**Figure 13:**
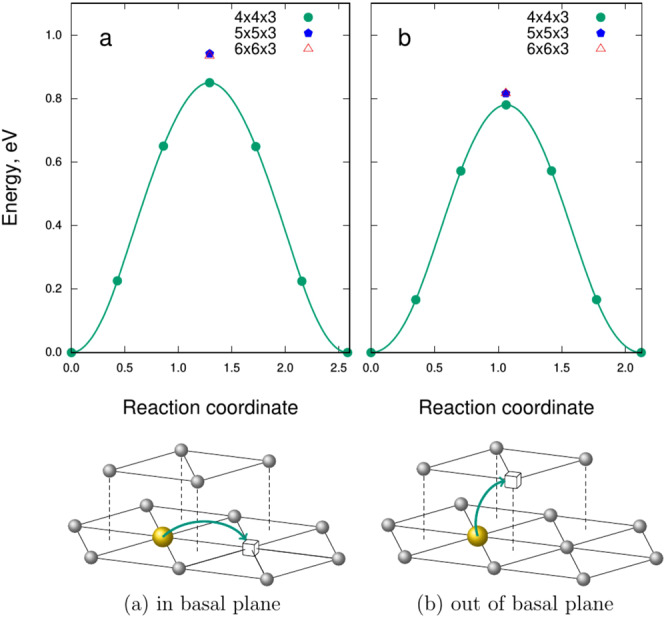
Energy landscape along iron and aluminum migration pathways: (**a**) jump within basal plane (**b**) jump out of basal plane.

However, our static *ab initio* calculations have shown that Fe, Al and Si atoms in beryllium prefer to stay close to the lattice site when another vacancy occasionally comes into its first coordination shell showing only moderate displacement towards the vacancy. Consequent NEB calculations have revealed that the position between two nearest neighbor vacancies (either within basal plane or in adjacent basal planes) appears to be a saddle point for the diffusion jump of Fe, Al and Si. As can be seen from the Fig. 13, the barriers for the jumps within (0.85 eV) and out of (0.78 eV) basal plane for substitutional iron are quite similar. On the contrary, diffusion of silicon is strongly anisotropic. The energy barrier for migration of aluminum is twice as low, i.e., about 0.4 eV both within and outside basal plane. The migration barrier for silicon (0.42 eV) is also lower than that for iron within basal plane, but is comparable (0.72 eV) with that for iron if silicon jumps into the adjacent basal plane. Contrary to anisotropic helium vacancy-mediated diffusion with significantly different migration barriers of 0.72 eV along and 1.19 eV outside of basal plane^1^, both diffusion of iron and aluminum is nearly isotropic at elevated temperatures. It should be noted that all migration barriers are comparable or even lower than those for vacancy diffusion (0.8 eV). It does not mean, however, that vacancy mediated solute diffusion is faster than that of vacancy. In the case of strong binding with solute, the rate-limiting step of solute diffusion is reorientation of a vacancy-solute pair, which requires vacancy jump around solute. This barrier is expected to be comparable with that for vacancy diffusion.

## Discussion

In the following section, we shortly summarize general knowledge on beryllium behavior after irradiation at different temperatures and emphasize new findings and outcomes obtained in this work.

The type of microstructure formed in beryllium under irradiation strongly depends on the irradiation temperature. Below 400–500 °C, interstitial loops nucleate first and then grow with increasing fluence, finally building together into dislocation network^24,25^. No bubble observations were reported in these works. On the contrary, at higher temperatures high number density of helium bubbles can be found, but no irradiation-induced loops are visible. The threshold temperature separating both microstructure types is not sharply defined being dependent on the irradiation dose. At low temperatures (below 400 °C), the weak mobility of helium-vacancy clusters sometimes can be compensated by a long irradiation time, so that bubble nuclei can slowly grow and become eventually visible in TEM^26^. This is the case for our samples irradiated at 370 °C and 440 °C. Although the irradiation temperatures in both cases are close to the threshold temperature, long irradiation time (~5 years) as well as high helium accumulation allowed bubble growth even at these relatively low temperatures.

### Dislocation loops

The circular feature shown in Fig. 10 is a segregation of iron and aluminum to the edge of an interstitial dislocation loop. It is commonly accepted that at high doses, irradiation-induced loops form dislocation networks, so that at irradiation doses above ~1 dpa individual loops can be observed only seldom. This raises the question why this loop is nevertheless visible. We suppose that it was immobilized by the large Al-Fe-Be precipitate as well as by several smaller ones (visible as darker spherical objects marked with arrows in Fig. 10b). During irradiation, both iron and aluminum atoms have segregated to the edge of the extra plane, where somewhat larger interatomic spacing than in the surrounding matrix is available. It can be also supposed, that segregation of the second phase prevents further adsorption of self-interstitials on the dislocation line, thus preventing its further growth. This effect is known as “point defect sink poisoning”. On the contrary, vacancies and gas atoms were adsorbed at the interface between the Al-Fe-Be phase and the matrix and participated in the growth of gas bubbles abundantly decorating all precipitates.

### Impurities in unirradiated material

As has been long established, some alloying elements can drastically affect the mechanical properties of beryllium at elevated temperatures and induce high-temperature brittleness, also termed as “hot shortness”^27^. In particular, aluminum and manganese tend to segregate to GBs as low-melting-temperature phases thus reducing the GB cohesion and, hence, ductility of beryllium above 450 °C. Therefore, it is important, that if present above their solubility limits, these impurities should be bound by iron and silicon to form intermetallic phases with higher melting temperatures. Formation of precipitates in beryllium after annealing was extensively investigated in the sixties of the last century^28,29^. However, up to our knowledge their behavior under irradiation was not reported yet.

Beryllium pebbles studied in this work contain several major impurities such as Fe, Al, Si, Mg (see Table 2). It is known that without irradiation, ternary Fe-Al-Be phase precipitates at GBs at temperatures in the range 650–850 °C, while at higher temperatures iron dissolves in beryllium matrix leaving free aluminum at GB. If iron concertation in the matrix exceeds its solubility limits (0.11 at% @800 °C^30^), it precipitates in the form of BeFe_11_ platelets. Aluminum solubility at this temperature is approximately ten times lower (0.07 at%@800 °C).

**Table 2:** Chemical composition of Ø 1 mm beryllium pebbles after irradiation in HIDOBE-01 [2].

Element	Concentration, wppm
Be	balance
Fe	1050
Al	376
Ni	153
Cu	113
Mg	116
Mn	104
Cr	102
Si	305
U	103
Ti	42
Zr	31
Au	20
V	19
O	153*
C	239*
N	< 20*

*Measured before irradiation.

### Second phase precipitates and segregation after irradiation

In our study, we have observed precipitates within grains only at two lowest irradiation temperatures (370 and 440 °C). No precipitates within grains were observed after irradiation at 560 °C, although without irradiation they should have been stable at this temperature. This suggests that stability of these precipitates decreases under irradiation and they dissolve at some temperature between 440–560 °C.

**Figure 14:**
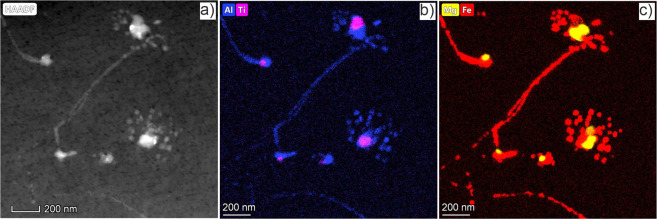
Images show a radiation-induced dissolution of complex precipitation. The HAADF image is represented in part (**a**) and in parts (**b–c**) the 2D distribution of the corresponding elements is represented.

In fact, dissolution due to the recoil atoms induced by neutron irradiation followed by reprecipitation of complex-phase precipitates was observed at 480 °C. Figure 14 shows two precipitates with sizes between 100–150 nm that are surrounded by numerous precipitates with sizes between 20–50 nm. The larger precipitates contain multiple phases of Al-Fe-Si-Cr-Ti-Mg-Mn-U elements, while the smaller precipitates contain a Fe-Al single phase. When a cascade of atomic collisions induced by neutron irradiation overlaps with a precipitate, its atoms can be ballistically knocked out into surrounding matrix leading to its dissolution^31^. During irradiation, this process is balanced by the growth of the precipitate due to a constant flux of solute atoms to the precipitate. According to the model presented by Nelson, Hudson and Mazey in ref. ^32^ smaller precipitates have a positive grow rate while the larger ones will dissolve due to their increasing surface area which increases the probability of cascade overlapping. This inverse coarsening is in contrast to the thermally activated Ostwald ripening of precipitates. As local solute atom concentration is increased in the direct vicinity of the precipitates by ballistic dissolution, a local supersaturation can lead to the nucleation of new precipitates in the its direct vicinity as it can be seen in the ref. ^33^. Dissolution and post-precipitation is probably possible only for the Fe-Al-Be phase with a low melting temperature.

Under irradiation, impurities segregate to point-defect sinks forming complex precipitates with multiple-phase composition (as exemplified in Fig. 9). The results of EELS and EDX studies presented in our previous publications^19,34^ suggest that these complex structures are beryllium intermetallic compounds. The post irradiation examination of HIDOBE1 campaign also confirmed the presence of complex beryllide particles with Fe/Al/Mn or Fe/Al/Mn/Cr composition^35^. It was found that the particles were located along the line dislocations. The electron diffraction pattern show that particles often Be_4_Al (FeCrMn) phase with a face-centered cubic structure^36^.

### Accumulation of tritium inventory

On the macroscopic scale, the major difference in the behavior of samples irradiated at low and high temperatures is manifested by volumetric swelling of beryllium. After irradiation at low temperatures, swelling is very small (about several percent) and is directly proportional to the generated helium content^9,37,38^. However, above 600 °C swelling of beryllium increases very rapidly with irradiation temperature^39^.

One of the major potential problems driving the investigations of the microstructure formed in beryllium under irradiation conditions close to those of fusion reactor blanket is accumulation of tritium inside about 300 tons of beryllium pebbles required for the operation of the future fusion reactor DEMO. If trapped inside beryllium, tritium bears a potential risk of burst release of this β-radioactive gas under accidental conditions. Moreover, accumulated in large amounts tritium would hinder radioactive waste processing and increase the safety requirements for storage of irradiated beryllium after the blanket end of life.

The amount of accumulated tritium depends on the strength, amount and capacity of traps. This immediately raises the question on where tritium is trapped in beryllium. In the following, we will discuss the observed microstructural changes with respect to their influence on possible tritium accumulation.

### Potential tritium traps

Vacancies are known to be efficient trapping sites of hydrogen isotopes in metals. According to our *ab initio* calculations, up to six tritium atoms can be trapped within one vacancy in beryllium^40^.

As predicted by *ab initio* simulations, helium bubbles are even stronger tritium traps with a binding energy of 1.8–2.0 eV^41^. Helium stabilizes di-vacancies and prevents dissolution of gas bubbles by evaporation of vacancies at high temperatures^2^. Our *ab initio* calculations have shown that, additionally, tritium atoms can be adsorbed at the bubble walls, as well as co-exist as gas molecules inside bubble. Experimental observation of formation of deuterium molecules in beryllium after D-implantation was reported by several authors^42^. Taken together, all these facts make helium bubbles primary candidates for tritium accumulation both in form of atoms adsorbed at the bubble walls as well as tritium molecules inside bubble interior.

#### Bubbles inside grains

TEM analysis shows that typically 8–12 nm large bubbles formed in the pebbles irradiated at 643 K (370 °C) with an unusual round shape (Fig. 2a). In isotropic materials like iron, such a shape is typical for small helium bubbles^43^, which often possess a spherical form without faceting. In anisotropic beryllium, round shape is rather surprising, as far as hexagonal prism-shaped bubbles are commonly observed^44^. In spite of their relatively large size of ~10 nm, bubbles might be overpressurized due to large quantities of helium gas generated (see Table 1). Similar disagreement between calculated and experimentally observed shapes of voids was found in magnesium too^45^.

Presently the reason for the discrepancy is unclear. Most probably some additional effects such as helium pressure, impurity segregation or, more likely, kinetic non-equilibrium character of bubble growth should be taken into account to improve modelling predictions.

#### Bubbles at grain boundaries

Larger bubbles with smaller number densities in comparison to those within grains were observed at GBs at all irradiation temperatures. The size of the bubbles increases and their number density decreases with irradiation temperature.

GBs are known as prevalent vacancy sources and sinks. Any local excess of vacancies is immediately redistributed along GBs, so that nucleation of pure voids at GB is impossible. Singh *et al*.^46^ concluded that nucleation of bubbles on GBs is associated with helium trapping there.

As voids tend to dissolve at GBs, it is helium which governs gas bubble growth at the GBs. GB bubbles should be close to equilibrium (i.e., gas pressure is defined by the surface tension) as far as the surplus vacancies can easily escape to the GB. The larger size of bubbles on the HAGB vs those grown inside grains can be tentatively explained by higher helium mobility along high angle GBs (HAGB) due to availability of excess volume there and lower density of nucleation sites (i.e. structural defects trapping helium) then in the grain interior.

### Denuded zones

The existence of denuded zones near GBs is commonly related to continuous annihilation of point defects as well as trapping of helium atoms at GB and respective reduction of the local concentrations of these species in its vicinity^47^. According to this model, nucleation of new bubbles is suppressed in these regions as far as both helium atoms and vacancies which are necessary for this process are deficient. The width of the bubble-denuded zone is determined by the vacancy and helium-vacancy cluster mobility and, hence, should increase with irradiation temperature. This behavior is in accordance with our experimental results showing widening of the bubble-denuded zone from about 120 nm at 643 K to 1200 nm at 923 K (see Table S1).

In addition, notably larger bubbles were observed just outside the denuded zone in comparison with those grown within the grain. Due to this fact, the region immediately adjacent to the void-denuded zone is commonly called a “peak zone”. This effect is long known and discussed on several occasions (see, e.g., ref. ^47^). The recent phase-field calculations of Millet *et al*.^44^ reproduced this effect by considering significantly higher mobility of interstitials versus that of vacancies leading to the difference in the widths of depleted zones for both species. This model emphasizes that the width of denuded zone is not universal, but depends among others on the mobility of the species considered.

Binding between aluminum and silicon with vacancy and its absence for iron imply certain consequences for their diffusion mechanism. Thus, aluminum and silicon will most probably diffuse as a solute-vacancy pair along vacancy concentration gradient. In contrary, iron will diffuse as a single atom moving against the gradient of vacancy concentration (so called inverse Kirkendall effect). In the latter case, segregation depends on the difference in diffusion rates of iron and beryllium in beryllium matrix. This behavior explains the enrichment or depletion of GB with these solute atoms under irradiation (vacancy concentration gradient points toward GB) or during thermal annealing (opposite direction of the gradient)^48^.

Binding of silicon, aluminum and helium solutes with vacancies provides additional vacancies and gas atoms necessary for nucleation and growth of gas bubbles. The excess of vacancies accumulates at the matrix-precipitate interface and contributes to formation of gas bubbles. This solute-vacancy binding provides an explanation for the abundant coverage of all Al-Fe-Be precipitates inside grain volume, as well as segregation of this phase along dislocation lines, with small gas bubbles. Segregations of Al-Fe-Be phase to dislocation lines were observed in this work (marked with arrows in Fig. 10b) as well as previously^35^.

**Figure 15:**
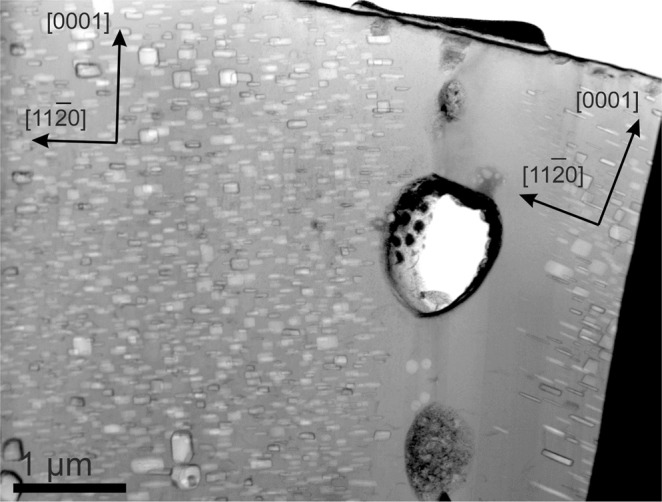
Grain boundary in pebble irradiated at 713 K. The size of denuded zone from both sides is quite different: 0.4 µm inside the left grain and 0.9 µm inside the right grain.

As we have shown before, interstitial helium diffuses also highly anisotropic with a preferential diffusion occurring within basal plane^1^. Both interstitial helium and helium-vacancy clusters migrate towards GBs thus contributing to the bubble-denuded zone formation. Our simulation results suggest that the width of the zones denuded by Fe-Al precipitates should be independent of the lattice orientation relative to the GB, while the width of the bubble-denuded zones should demonstrate such dependence due to pronounced anisotropy of helium diffusion. Our TEM studies seem to be in line with this conclusion (see Fig. 15). However, more statistics is necessary to confirm it without a doubt.

Denuded zones with thicknesses of 150 nm and 300 nm free of Al-Fe-Be precipitates were observed for 643 K (370 °C) and 713 K (440 °C), respectively. Bubble-denuded zones being 100 nm at 643 K (Fig. 8a), 250 nm inside the lower, 120 nm inside the upper grain at 713 K (Fig. 6) are notably smaller than the precipitate-denuded zones at the same irradiation temperature. As was discussed in the previous section, commonly accepted reason for denuded zone formation is depletion of vacancy as well as helium or iron concentrations near GBs. Width of the depleted zone depends on the mobility of corresponding species and, hence, is different for Fe-Al-Be precipitates and helium bubbles.

## Conclusions

Microstructural changes induced by neutron irradiation in beryllium were studied, both experimentally and by application of the first principles methods. Targeted preparation of TEM lamellas cut across various GBs was performed from beryllium irradiated at 643, 713, 833, and 923 K up to an accumulation of 3600–5900 appm helium. Present investigations were focused on the characterization of gas bubbles located inside grains and at the GBs as well as on the study of spatial distribution and composition of secondary phase precipitates.

Abundant formation of helium bubbles within grains was found at all irradiation temperatures being consistent with previous literature. Their shape is spherical at 643 K and changes to the flat hexagonal prism at higher irradiation temperatures. Bubble size strongly increases with irradiation temperature. Apparent swelling estimated from the TEM data is reaching ~9% at 923 K. Bubbles significantly larger than those observed within grains, however with smaller number density, are found along GBs.

Precipitation of Fe-Al-Be phase within grains, along dislocation lines and GBs as well as segregation of iron and chromium at GBs were revealed. Denuded zones free of bubbles and Fe-Al-Be precipitates with width increasing with irradiation temperature were observed. The width of denuded zones is different for the bubble-free and the precipitate-free zones.

Our *ab initio* calculations reveal notable binding of silicon and aluminum solutes with vacancies at various distances. On the contrary, the iron atom practically does not deform beryllium lattice and repels vacancies. These results suggest different diffusion mechanisms for these solutes. Silicon and aluminum may diffuse as a solute-vacancy pair, while iron migrates as single atom against gradient of vacancy concentration. In addition, our calculations have shown, that Al and Fe solutes diffuse nearly isotropic in beryllium, in contrary to silicon and helium, which diffuse faster along basal planes. We conclude that anisotropic diffusion of helium should be reflected in the different width of bubble-denuded zones depending on GB orientation relative to basal planes in particular grains. Conversely, due to isotropic diffusion of Al and Fe the precipitate-denuded zones should not demonstrate such dependence.

## Experimental and simulation methods

### Experiment

The investigated beryllium pebbles with a diameter of about 1 mm were fabricated by NGK Insulators Ltd., Japan using the rotating electrode method^49^. Their chemical composition is given in Table 2^50^ and the parameters of irradiation, performed in the High Flux Reactor (HFR) Petten within the HIDOBE 2 campaign^4^, are shown in Table 1. The irradiation temperatures within the rigs, which were continuously controlled during irradiation with thermocouples, showed large variations. Therefore, average temperatures are used in this work, which are ca 20–50 °C away from the target temperatures commonly reported in the previous publication^51^.

For the preparation of the TEM samples, irradiated pebbles were embedded into epoxy resin and mechanically polished to obtain a metallographic cross section suitable for optical microscopy. This step provided us with a first glance into the microstructure of the irradiated material. Obtained cross-sections were examined in optical microscope and regions of interest were selected. High-angle grain boundaries were identified by chains of bubbles or different coloring in polarized light distinguishable in optical microscope. The major aim of this work is to study microstructural changes both at GBs and inside grains. To resolve other grain boundaries and gather quantitative information we have acquired EBSD maps at the Centre for Fusion Energy, Culham, UK. TEM lamellae were cut from the chosen areas using Focused Ion Beam (FIB) Auriga and placed on molybdenum grids. The part of lamella transparent for electron beam has typically a size of 4–8 µm. TEM imaging and most analytical mapping were performed using FEI Tecnai F20 microscope. Several elemental maps were obtained using Super X-EDS system of Talos F200X TEM (Thermo Scientific). In colored figures presenting EDX results overlapping of 2D maps of individual elements is presented.

### Simulation methods

The first principles calculations performed in this work were based on the density functional theory as implemented in the simulation program package VASP^52^. Projector augmented-wave (PAW) potentials^53^ with two (s2p0), eight (d7s1) and three (s2p1) electrons were selected for beryllium, iron, and aluminum, respectively^54,55^. The generalized gradient approximation (GGA) of Perdew and Wang^56^ was used for the calculation of the exchange-correlation energy.

During relaxation of defect configurations, both volume and shape of the simulation cells were fixed, while coordinates of all atoms were allowed to relax freely (ISIF = 2). The Fermi broadening method with a smearing of 0.2 eV and a plane wave energy cut-off of 750 eV were chosen after testing convergence of the results. The convergence with respect to the k-point mesh is reached at 11 × 11 × 11. As a trade-off between accuracy, speed and required memory, the gamma-centered k-point meshes shown in Table S1 were used. To test convergence of the obtained results with increasing simulation cell size, simulation cells containing various number of atoms (90–216 atoms) were used. Self-consistent electronic loop is aborted when the change in the total energy is less than 10^−6^ eV (tag EDIFF). Ion relaxation is stopped whenever all forces on atoms are below 10^–3^ eV/Å (tag EDIFFG).

The binding energy of a solute atom with vacancy ${E}_{b}^{S-{Vc}}$ was calculated as follows:

1 $${E}_{b}^{S-Vc}=({E}^{S}+{E}^{Vc})-({E}^{S-Vc}+{E}^{Be}),$$

where ${E}^{S-{Vc}}$ is the energy of configuration containing one solute and a vacancy at certain distance from it, ${E}^{{Be}}$ is the energy of perfect beryllium lattice, ${E}^{S}$ and ${E}^{{Vc}}$ are the energies of configurations containing single solute and single vacancy, respectively. Binding energy defined by Eq. 1 is positive in the case of attraction between solute and vacancy.

The energy profiles for two migration pathways considered in the paper were determined using nudged elastic band method (NEB)^57^ as implemented in the VTST tools patch^58^ for the VASP code. Due to higher computer time demands in comparison with static relaxations, the NEB calculations were performed for simulations cells containing 96 beryllium atoms (4 × 4 × 3 unit cells) only and using five intermediate images. However, the saddle point positions, which are stable configurations because of force cancellations due to symmetry, were crosschecked for convergence using larger simulation cells. The height of the barriers changes by less than 0.1 eV when the simulation cell size is increased (see Table S1). For the NEB calculations, slightly relaxed set of parameters was employed: ENCUT = 450 eV, the change in total energy should be below 10^−6^ eV for aborting electronic loop, while ion relaxation stops after all forces drop below 10^−2^ eV/Å. Quick minimization algorithm (IBRION = 3) from the VTST package was used for ion movement. We applied VTST scripts for post-processing and plotting the results of NEB runs.

## Supplementary information

Supplementary Information.
