# Author Correction: New insights into microstructure of irradiated beryllium based on experiments and computer simulations

**DOI:** 10.1038/s41598-020-76736-5

**Published:** 2020-11-11

**Authors:** M. Klimenkov, P. Vladimirov, U. Jäntsch, V. Kuksenko, R. Rolli, A. Möslang, N. Zimber

**Affiliations:** 1 Institute for Applied Materials - Applied Materials Physics (IAM-AWP), Karlsruhe Institute of Technology (KIT), Hermann-von-Helmholtz-Platz 1, 76344 Eggenstein-Leopoldshafen, Germany; 2 Culham Centre for Fusion Energy, Culham Science Centre, Abingdon, OX14 3DB Oxfordshire UK; 3 Institute for Applied Materials - Materials and Biomechanics (IAM-WBM), Karlsruhe Institute of Technology (KIT), Hermann-von-Helmholtz-Platz 1, 76344 Eggenstein-Leopoldshafen, Germany

Correction to: *Scientific Reports* https://doi.org/10.1038/s41598-020-64654-5, published online 15 May 2020

The original version of this Article contained multiple errors.

The incorrect image was used for Figure 13.

Furthermore, there was a typographical error in the legend of Figure 14.

“Images show a radiation-induced dissolution of complex precipitation. The HAADF image is represented in part (**a**) and in part (**b–i**) the 2D distribution of the corresponding elements is represented.”

now reads:

“Images show a radiation-induced dissolution of complex precipitation. The HAADF image is represented in part (**a**) and in part (**b–c**) the 2D distribution of the corresponding elements is represented.”

Finally, a Supplementary File containing Tables S1 and S2 was omitted from the original version of this Article.

These errors have now been corrected in the HTML and PDF versions of the Article.

