# Supplementary material for: New insights into microstructure of irradiated beryllium based on experiments and computer simulations
Source: Sci Rep. 2020 May 15;10:8042. doi: 10.1038/s41598-020-64654-5 (PMC7229013; doi:10.1038/s41598-020-64654-5)
Supplement: Supplementary file 1 — Supplementary Information. [file 41598_2020_64654_MOESM1_ESM.docx]

New insights into microstructure of irradiated beryllium based on experiments and computer simulations

M. Klimenkov^1*^, P. Vladimirov^1^, U. Jäntsch^1^, V. Kuksenko^2^, R. Rolli^3^, A. Möslang^1,^ N. Zimber^1^

^1^Karlsruhe Institute of Technology (KIT), Institute for Applied Materials - Applied Materials Physics (IAM-AWP), Hermann-von-Helmholtz-Platz 1, 76344 Eggenstein-Leopoldshafen, Germany

^2^Culham Centre for Fusion Energy, Culham Science Centre, Abingdon, Oxfordshire, OX14 3DB, United Kingdom

^3^Karlsruhe Institute of Technology (KIT), Institute for Applied Materials - Materials and Biomechanics (IAM-WBM), Hermann-von-Helmholtz-Platz 1, 76344 Eggenstein-Leopoldshafen, Germany

Corresponding author: Michael Klimenkov

Telephone number: +49 721 608 22903

Fax number: +49 721 608 24567

E-mail address: ***michael.klimenkov@kit.edu***

Supplementary:

Table S1. Parameters of microstructural objects (gas bubbles, denuded zone, etc.) in beryllium pebbles irradiated in HIDOBE-02 (GB – grain boundary).

| <T_irr_>, K | <D>, nm | Standard deviation σ_D,_ nm | <H>, nm | <D>/<H> | D of bubbles on GBs, nm | Width, nm | | N, m^-3^ | L, nm | Swelling, % |
| --- | --- | --- | --- | --- | --- | --- | --- | --- | --- | --- |
|  |  |  |  |  |  | Bubble-denuded zone | Precipitate-denuded zone |  |  |  |
| 643 | 11.5 | 1.6 | 11.5 | 1 | 5-20 | ~100 | 150 | 1.9·10^22^ | 46 | 1.7 |
| 713 | 29 | 6 | 11.8 | 2.5 | 70-  200 | 200-240 | 300 | 2.0·10^21^ | 98 | 3.2 |
| 833 | 67 | 15 | 19 | 3.5 | ⎯ | ⎯ | ⎯ | 7.2·10^20^ | 138 | 6.5 |
| 923 | 96 | 33 | 25.5 | 3.8 | 100-  1200 | 700-1200 | ⎯ | 4.5·10^20^ | 161 | 8.7 |

Table S2. Binding energies of iron, aluminum and silicon solutes in beryllium with vacancy at various distances.^[[1]](#footnote-1)^

| NN | Dist, Å | Config | Binding energy of Fe, eV | | | Binding energy of Al, eV | | | Binding energy of Si, eV | | |
| --- | --- | --- | --- | --- | --- | --- | --- | --- | --- | --- | --- |
| #atoms |  |  | 96 | 150 | 216 | 96 | 150 | 216 | 96 | 150 | 216 |
| k-points |  |  | set1 | set2 | set3 | set1 | set2 | set3 | set1 | set2 | set3 |
| 1 | 2.21 | 1NNn | -0.308 | -0*.*505 | -0*.*416 | 0.703 | 0.584 | 0*.*699 | 1.112 | 0.959 | 1.037 |
| 2 | 2.28 | 1NNb | -0.320 | -0*.*424 | -0*.*385 | 0.619 | 0.497 | 0*.*541 | 1.041 | 0.876 | 0.868 |
| 3 | 3.17 | 2NNn | -0.020 | -0*.*111 | -0*.*050 | 0.248 | 0.121 | 0*.*228 | 0.565 | 0.392 | 0.464 |
| 4 | 3.57 | 3NNn | 0.252 | 0*.*160 | 0*.*195 | 0.304 | 0.193 | 0*.*300 | 0.529 | 0.407 | 0.476 |
| 5 | 3.90 | 2NNb | -0.012 | -0*.*142 | -0*.*065 | 0.252 | 0.181 | 0*.*225 | 0.521 | 0.319 | 0.384 |
| 6 | 3.92 | 4NNn | -0.059 | -0*.*137 | -0*.*111 | 0.269 | 0.141 | 0*.*248 | 0.510 | 0.337 | 0.396 |
| 7 | 4.23 | 5NNn | -0.065 | -0*.*157 | -0*.*111 | 0.205 | 0.096 | 0*.*198 | 0.448 | 0.312 | 0.377 |
| 8 | 4.48 | 3NNb |  | -0*.*149 | -0*.*069 |  | 0.095 | 0*.*192 |  |  |  |

1. K-points meshes used: set1 - 11×11×7, set2 - 11×11×11, set3 - 9×9×11 [↑](#footnote-ref-1)
